# 肿瘤基因组图谱泛癌分析计划

**DOI:** 10.3779/j.issn.1009-3419.2015.04.02

**Published:** 2015-04-20

**Affiliations:** 100071 北京，中国人民解放军军事医学科学院附属医院肺部肿瘤科 Department of Lung Oncology, Afliated Hospital of the PLA Military Academy of Medical Sciences, Beijing 100071, China

**Keywords:** 肿瘤, 治疗, 肿瘤基因组图谱, Neoplasms, Therapy, The Cancer Genome Atlas

## Abstract

由于起源部位、细胞类型、基因突变形式的不同，肿瘤可表现出不同的形态。这些因素同时影响肿瘤的治疗效果。虽然已经证实许多基因的改变直接导致了表型的改变，然而，许多肿瘤谱系中复杂分子机制依然未完全阐明。因此，肿瘤基因组图谱（The Cancer Genome Atlas, TCGA）研究网络分析了大量人类肿瘤，以期发现DNA、RNA、蛋白质和表观遗传学水平的分子改变。含丰富数据的结果给我们提供了一个机会，对整个肿瘤谱系的共性、个性及新观点形成一个整体描绘。基因组图谱泛癌计划最先比较了12种肿瘤类型。对不同肿瘤分子改变及其功能的分析，未来会告诉我们如何将有效的治疗方法应用于类似基因表型的肿瘤。

## Abstract (en)

Cancer can exhibit different forms depending on the site of origin, cell types, the different forms of genetic mutations which also affect cancer therapeutic effect. Although many genes have been demonstrated to change a direct result of the change in phenotype, however, many cancers lineage complex molecular mechanisms are still not fully elucidated. Therefore, The Cancer Genome Atlas (TCGA) Research Network analyzed a large human tumors, in order to find the molecular changes in DNA, RNA, protein and epigenetic level, The results contain a wealth of data provides us with an opportunity for common, personality and new ideas throughout the cancer lineages form a whole description. Pan-cancer genome program first compares the 12 kinds of cancer types. Analysis of different tumor molecular changes and their functions, will tell us how effective treatment method is applied to a similar phenotype of the tumor.

在2014年9月中国临床肿瘤学会（Chinese Society of Clinical Oncology, CSCO）会议上，吴一龙教授作了一项重要发言，核心内容是“-OMA”的概念，即多种不同类别多种肿瘤的分享相同基因而且对其相关抑制剂治疗有效，给相关领域的同道同仁以很大启发。本文以下内容就是最好的关于不同肿瘤基因水平的系列启示，以此与同行共勉。

## 1 单个肿瘤类型的分子描述

如今，肿瘤作为一种基因病已达成共识。早期，通过已知阳性选择系统，对肿瘤遗传物质进行功能分析，识别了大量癌基因^[1-3]^；通过分析杂合子的缺失识别了一部分肿瘤抑癌基因^[4]^。最近，系统肿瘤基因组计划，应用新技术对特定肿瘤类型进行了分析，这其中包括肿瘤基因组图谱计划。这种特异性技术鉴定了新的导致肿瘤功能改变的驱动基因^[5-7]^，建立了新的分子亚型^[8-13]^，根据基因组学、转录组学、蛋白组学的变化识别了新的生物标记物。一些标记物已用于临床^[14, 15]^。比如，乳腺导管癌也是个典型例子，其不同亚型（luminalA、luminalB、HER2、基底样）临床特征完全不同。靶向治疗*BRAF*突变后，转移性黑色素瘤预后得到改善^[16]^。大部分肺癌的驱动基因位点（如EGFR、ALK、c-MET、KRAS、ROS1等）变化发现后，可靶向治疗的肺癌类型正在不断增加^[17, 18]^。形成癌基因的大量过程也同样得到了识别。Chromothripsis^[19]^和chromoplexy^[20]^，包括与基因重排高突变率相关的大量基因座染色体碎裂、重排^[21]^，为我们提供了观察肿瘤进化的视野^[22]^。

## 2 跨越不同肿瘤类型的分析

肿瘤样本数据的增加增强了我们探测分析肿瘤分子缺陷的能力。例如，不同肿瘤细胞重复性事件中，染色体微小区域的扩增与删除，可以精确描绘出驱动基因。大样本量DNA测序揭示了一系列周期性基因变化（突变、扩增、删除、易位、融合和其他结构变化），这些不同肿瘤类型中的基因改变，有的已经明确，有的还未有人发现^[23]^。然而，样本中基因变化分布的“long tail”也被揭示^[24]^。确实，TCGA一部分样本的基因变化类型与其他的不同。虽然每个肿瘤的表型与其他肿瘤明显不同，但每个肿瘤的分子变化，最终都汇合到共同的分子通路中。再如，通过整合不同肿瘤类型，少数体细胞突变可被鉴定为驱动基因，这样可以改进检测手段。如蛋白编码区的热点突变，可以发现潜在的药物治疗靶点。

判断少数变化位点是驱动子，或仅仅是辅助作用，或无临床活性，需要进一步功能评估和对其他肿瘤进行分析。识别每种肿瘤更多的驱动子突变及获得性缺陷，无疑会促进个体化治疗。靶向现已明确的140个驱动基因^[23]^进行治疗，虽然看起来可行，然而却是使人畏缩的。在不远将来，对数千个突变位点设计一次性治疗方法则更具有挑战性。

虽然经过几十年的研究，发现了一些重要的普遍规律^[25, 26]^，大多数对分子、病理、肿瘤临床特征的研究都以肿瘤类型的划分为依据^[27]^。只要看一下大多数肿瘤中心肿瘤科的指导原则，对肿瘤的医疗原则和手术原则都是以组织器官起源作为分类依据的。这种框架已经存在很长时间，但分子层面的分析认为这种观点存在问题。来自不同器官的肿瘤存在着许多共同特征，相反，来自相同器官的肿瘤有许多不同。

现已识别出来自不同器官肿瘤亚型的重要相似性。如TP53突变驱动高度恶性卵巢肿瘤、恶性子宫内膜癌、基底样乳腺癌，这些肿瘤有广泛相同的癌基因转录活化信号通路。同样，恶性胶质瘤、胃癌、恶性子宫内膜癌、膀胱癌和肺癌中发生了*ERBB2*/*HER2*的突变和（或）扩增。这些结论，可使这些肿瘤像治疗HER2扩增的乳腺癌一样，对靶向HER2的治疗有反应。其他肿瘤间的共性如恶性卵巢肿瘤和基底样型肿瘤中遗传性的体细胞BRCA1/2信号通路的失活，结直肠癌和子宫内膜癌内肿瘤相关基因微片段的不稳定，在结肠癌和子宫内膜癌中，新识别的POLE介导的超高突变率的亚型^[12, 28, 29]^。相反，来自不同器官，具有相同基因变化的肿瘤表现不同。主要的例子是NOTCH，其在肺鳞癌、头颈部癌^[30]^、皮肤癌^[31]^和子宫颈癌^[32]^中失活，但在液体瘤中通过突变而活化^[33]^。

这些例子表明形成跨越肿瘤类型的广阔视野的重要性，而不是根据组织病理学诊断，共同的分子特征将使一种肿瘤的病因学及治疗学新发现应用于另一种肿瘤。更重要的是，对数据全面的解释将帮助我们认识不同组织间突变导致的结果的差异。发生率低的肿瘤，如小儿恶性肿瘤，受益颇多。

当今世界我们对主要肿瘤的分子特征比几年前了解得更多。但一旦肿瘤转移，它依然无法治愈。对组织、器官、转移定植部位分子特征的整合是否会改善患者预后，还需要时间检验。但这种新方法占有优势。因此，泛癌计划的目标是识别分析决定肿瘤谱系的基因型和表型产生共性和差异性的基因变化。以下这份报告概括和介绍了这项计划。

## 3 泛癌计划

为了分析不同类型、组织起源肿瘤的共性、差异以及新课题。TCGA于2012年10月26日-27日在圣克鲁兹，加州举行的会议中发起了泛癌计划。泛癌计划是一个协同计划，目的是整合基于不同肿瘤类型，不同平台的TCGA数据，同时分析解释这些数据。在计划开始两月后，一篇名为“freeze”的文章发表，基于首批12种TCGA类型，每种肿瘤类型通过六种不同的基因组学、表观遗传学、转录组学和蛋白组学的平台进行分析。自此，整合的数据由一组多数来自TCGA研究员的研究人员进行质量控制和统计学分析。

泛癌计划基于一个分析框架，未来将包括新的肿瘤类型和来自TCGA和其他相似数据库的数据。现如今已经有很多同仁的合作努力，如小儿肿瘤邻域的合作研究（Therapeutically Applicable Research to Generate Effective Treatments, TARGET）、成人肿瘤研究计划（the nternational Cancer Genomics Consortium, ICGC）以及世界其他地方的小型计划。一个关键组成部分是CTD项目（Cancer Target Discovery and Development）以及ICBP（Integrative Cancer Biology Program）项目。分别研究个体基因突变的功能定位，阐明其中的通路和网络。

超越单个肿瘤类型视野的肿瘤生物学存在一些问题，主要包括以下几个方面。

泛癌计划通过整合12类肿瘤类型以得到大量样本，统计数据的增加能否使驱动位点突变与其他位点突变区别。整合后的泛癌计划数据，使我们能识别新的驱动基因位点。新计算机辅助方法通过分析肿瘤复制时间和与背景基因突变率相关的肿瘤表达，可使我们识别频繁突变的基因。同时排除许多针对单一肿瘤类型研究计划的假阳性和假阴性^[34]^。将来，通过识别重复出现的突变和排他性结果，有利于区分驱动基因突变和普通基因突变。

哪种组织水平是肿瘤基因结构变化的基础？通过改进的方法，对大块染色体区段的结构分析可以改善我们识别多个重点区域基因调节因子和表观遗传学调节因子的能力，这些调节因子是通过对比不同肿瘤类型的数据发现的。组织相关类型已被用于检测全基因组复制的频率和时段^[35]^。

通过对不同组织的所有突变事件进行整体考虑，哪种通路是关键通路并且能潜在活化呢？如何获得像染色体结构重组这样的新突变类型：①收集不同肿瘤类型的低频率事件；②整合如突变、拷贝数改变、表观遗传学沉默等事件；③运用多种方法验证新预测的驱动基因；④运用基因网络和通路整合各种基因。

增加样本量能促进对同时发生相互各异的基因变化的分析吗？能帮助我们区分驱动基因和普通基因吗？在对不同组织、不同通路致癌机理的研究中，对基因组学、表观基因组学的狭窄视野会揭示出一幅虚假的图景^[36]^。

分子亚型能区分疾病中组织特异和组织非特异的成分吗？对表观基因组学、转录组学和蛋白组学的分析提示不同组织类型对肿瘤细胞通路的影响。另外，基因表达图景证实了组织依赖的通路改变，其中包括对肿瘤相对重要的一百多种蛋白。对所有肿瘤类型的研究可使组织特异肿瘤信号从数据库中剔除。有趣的是，从DNA微阵列基因表达数据库中剔除的信号表明，免疫间质的效应跨越了肿瘤类型的界限。再次，不同谱系共同事件在泛癌计划中变得明显^[37]^。如激素依赖性乳腺癌、卵巢肿瘤、子宫内膜癌以及头颈部、肺部、子宫颈和膀胱肿瘤的常见鳞状化生。

在两种谱系中都活化的事件可以增加发现靶向特异治疗方法的范围。对计算机处理方式的整体评估可以揭示一些方法原则，使用跨不同类型肿瘤的整合信息预测患者结果。

## 4 泛癌计划受到的限制

现在，对不同数据库的整合不可避免的对泛癌分析造成了限制。关键问题在于对不同平台的数据进行的整合，或在技术改进时对数据平台的升级。例如，对更高密度DNA甲基化序列的转化，使用不同的外显子研究技术，RNA序列和基于微阵列的RNA特征，用于反相蛋白质阵列的抗体数量和质量的增加。已有一系列减少不同平台之间误差的方法。然而，有更多工作需要去做，以建立一套减少误差的方法，同时可以保存有用生物学信号。

不同肿瘤的临床数据的种类和质量差异很大。这些差异限制了建立通用统计信息、组织病理特征、行为环境和临床结果的能力。如由于诊断率低，卵巢肿瘤的生存率数据比较健全。但由于乳腺和子宫内膜肿瘤存活时间较长，其数据库还不成熟。只有当一些数据被认为相关时，才会采集这些数据（例如，肺部、膀胱、头颈部肿瘤的吸烟史）。已证实了一些实体肿瘤如头颈部肿瘤、子宫颈肿瘤、卡波西肉瘤和肝细胞癌与病毒有关。但是，只有少数肿瘤有感染因素登记，所以感染的病因学统计难以建立（作为一项常规数据统计）。最后，不同肿瘤有不同分类系统，难以建立共同的分级分期系统。这些挑战更反映出现今以组织和器官分类的临床实践特点。

从统计学上说，必须小心处理不同肿瘤对比得到的样本数据，以免得到假阴性率高的发现（如剔除重要突变）或假阳性率高的发现（如混淆从一种肿瘤得到的假阳性结果）^[34]^。

稀有事件不应该被疾病相关事件掩盖。肿瘤谱系在所研究的基因变化和基因表达描述中发挥重要作用，看似相同的事件，却有不同的结果，如相同的基因和扩增子。同样，为准确探测肿瘤趋势的新方法需要准确依据不同组织起源的肿瘤突变率、拷贝数改变，以及其他基因背景和表观遗传学背景同时发生事件的趋势。

虽然有以上挑战，泛癌计划所发表的文章表明其在分子水平研究肿瘤生物学的共性和差异所作的努力。同样，今后泛癌计划的主要问题依然存在，用于比较不同肿瘤的技术会随着实践、时间和将来的合作而改进。

## 5 今后的方向

泛癌计划是第一个协同研究不同肿瘤分子图景的，尤其是对多种肿瘤的大量研究。进一步增加每种肿瘤的样本量和肿瘤的种类无疑会使我们检测特异肿瘤样本稀有驱动基因的能力增强。但这种进步最终来自对不同类型的详细分析、与之相关的临床预后、实验验证、临床试验来检验假设。像激光显微切割、细胞分类等技术可使我们区分Omic信号是恶性或来自正常细胞。组织学描述、基于质谱分析的蛋白质分析、单个肿瘤细胞异质性的反卷积分析法等方法可以给我们提供新的重要的信息。对肿瘤来源细胞的追踪可使我们区分肿瘤的共性和差异性。克隆水平和不同肿瘤间单个分子的比较可能会揭示不同肿瘤的联系。对切除的瘤体、原位复发的瘤体、转移的瘤体的纵向研究会通过合作取得进展。在此之前只限于原发肿瘤的研究，且缺乏对治疗效果的信息。原发肿瘤的特征随着转移可能会变化，特别是骨肿瘤和脑瘤。泛癌计划对转移的分析将绘制出转移瘤与原发瘤及正常组织间的关系。揭示侵袭和定植的基本规律。

随着对单个肿瘤分子高分辨率监视的实现，泛癌计划的分析能力会增强。现在基因测序的价格已下降，下一个泛癌计划项目将能够分析各个肿瘤全基因组序列。全基因组分析会补充非编码区的突变过程，这部分现在还未研究。扩展分析主要集中在对启动子、增强子断裂，非编码RNA突变，肿瘤进化中像反转录转座子、病毒等易变的内源性和外源性DNA元件的分析上。全基因组测序将生成基因关联研究的背景，以使内在易染病体质和特定肿瘤联系起来。基于相关通路和网络的研究方法将和来自宝贵数据的治疗机会相结合。后续试验将集中在序列的功能研究以及新发现与治疗之间的关系上。

## 6 结语

像泛癌研究计划这种对不同肿瘤的综合研究最终无疑会影响临床决策。我们希望发现能用于临床的新型治疗药物，或许是新的适应性强的、跨越不同肿瘤界限的、基于生物标记物的临床试验。为实现此目标，泛癌TCGA数据库的一部分已公开。虽然协同研究来自不同数据库的数据依然是个挑战，这些数据库包含从不同形式、不同层面分析肿瘤的资源。

关键挑战是设计基于分子标记的不同组织肿瘤亚型相关的临床试验策略。最近对跨多平台肿瘤细胞的药理学试验分析显示共同的基因变化可以预测不同谱系细胞对治疗的反应^[36-39]^。基于生物标记物的临床设计可增强其统计学意义，大大减少临床实验的规模、资金和周期。

供科学界去挖掘和探索的Omic数据库在数量和规模上在快速扩张，为研究肿瘤发病原因的计算机工具也变得更强大。关键是认识到整个计划的全部潜力会随着时间和努力的投入而实现。同时，TCGA泛癌计划发表的论文代表了对肿瘤研究新领域的贡献。
